# Zoonotic transmission and genetic diversity of *Leishmania major* in Shiraz, Iran: An integrated entomological, clinical, and molecular study

**DOI:** 10.1016/j.parepi.2026.e00487

**Published:** 2026-02-25

**Authors:** Kourosh Azizi, Saeed Shahabi, Bahador Sarkari, Qasem Asgari, Aboozar Soltani, Mohsen Kalantari, Azim Paksa, Sorna Dabaghmanesh

**Affiliations:** a Research Center for Health Sciences, Institute of Health, Department of Biology and Control of Disease Vectors, School of Health, Shiraz University of Medical Sciences, Shiraz, Iran; b Department of Parasitology and Mycology, School of Medicine, Shiraz University of Medical Sciences, Shiraz, Iran

**Keywords:** Disease, *Leishmania*, Phylogeny, Tourism, Sequencing, Iran, ITS2, kDNA

## Abstract

Cutaneous leishmaniasis (CL), endemic in Shiraz, Iran, is caused by *Leishmania major* (zoonotic cutaneous leishmaniasis, ZCL) and *L. tropica* (anthroponotic cutaneous leishmaniasis, ACL). This study investigated transmission drivers, including sand fly vectors, zoonotic and environmental factors, and the genetic diversity of *L. major*. A total of 1029 sand flies were collected indoors and outdoors in Shiraz from August to October using sticky traps. Samples from patient lesions (*n* = 30) and pooled female sand flies (*n* = 40 pools) were examined by microscopy and screened by PCR targeting kDNA and ITS2 genes. Phylogenetic and haplotype analyses were conducted based on ITS2 sequences.

The sand fly fauna was dominated by *Phlebotomus papatasi* (53.8%) and *Ph. sergenti* (38.7%). PCR detected *L. major* DNA in 80% of *Ph. papatasi* pools and both *Leishmania* species in *Ph. sergenti* pools; however, molecular detection alone does not confirm vector competence. All patient samples were confirmed positive for CL. ITS2 sequencing identified 22 haplotypes among *L. major* strains, 20 of which were detected in Iran, indicating relatively high haplotype diversity (Hd = 0.74). A dominant ancestral haplotype was shared across Asia, Africa, and Europe.

These findings suggest that ZCL transmission in Shiraz is likely associated with peridomestic sand flies (*Ph. papatasi* and *Ph. sergenti*) and environmental conditions that may facilitate transmission. Iran accounted for 91% of the identified haplotypes in this dataset, highlighting its substantial contribution to regional ITS2 diversity. Continued vector surveillance and improved environmental management may support more effective control strategies.

## Highlights

• Iran hosts 91% global “*L. major*” haplotypes (20/22, Hd = 0.74), signaling an ancient evolutionary hotspot with pandemic risk.
• Peridomestic sand flies (*Ph. papatasi*: 86% indoor) show 72.5% Leishmania infection, may contribute to garbage/sewage/animal cohabitation.
• *Ph. sergenti* tested PCR-positive for both *L. major* and *L. tropica*, indicating exposure to both parasites but not confirming vector competence for *L. major*
• Ancestral *L. major* haplotype (Hap1) spans Asia/Africa/Europe, confirming Iran as a global dispersal reservoir.
• It seems that vector control and sanitation to halt strain emergence amid high genetic are needed

## 1 Introduction

Leishmaniasis is a neglected tropical disease (NTD) caused by protozoan parasites of the genus *Leishmania* and transmitted through the bites of infected female phlebotomine sand flies. The disease presents in several clinical forms, including cutaneous leishmaniasis—classified as anthroponotic (ACL) and zoonotic (ZCL)—as well as visceral (VL), mucocutaneous, and diffuse cutaneous leishmaniasis (DCL). These clinical forms are endemic in many tropical and subtropical regions, including Iran (Azizi et al., 2016; Azizi et al., 2013; Azizi et al., 2012a, Azizi et al., 2012b, Azizi et al., 2012c; Davoodi et al., 2022; Najafi et al., 2021; Rezaei et al., 2020).

According to the World Health Organization (WHO), the annual the annual incidence of ACL and ZCL is estimated to range from 0.7 to 1.3 million cases worldwide. Although rarely fatal, the disease causes disfiguring skin lesions and scars—particularly on the face—which can lead to significant social stigma and negative psychological effects, especially among women. The Middle East reports approximately 80% of the world's CL cases, making it a major public health concern in countries such as Afghanistan, Pakistan, Iran, and Iraq (WHO, 2020).

There are cutaneous and visceral forms of the disease in Iran. The spread of *Leishmania* vectors and reservoirs has increased in recent years. This rise has led to the formation of multiple ACL and ZCL foci in the central, western, and southwestern regions of the country. Fars province, is one of the major centers for cutaneous and visceral leishmaniasis in Iran (Azizi et al., 2012a, Azizi et al., 2012b, Azizi et al., 2012c; Davoodi et al., 2022; Ghatee et al., 2020; Gigloo et al., 2018; Kalantari et al., 2025; Rezaei et al., 2020; Shahabi et al., 2023, Shahabi et al., 2024).

The spread of leishmaniasis is likely driven by multiple factors, including vector and reservoir abundance, habitat destruction, climate change, and socio-cultural conditions. The primary vectors globally are sand fly species of the genera *Phlebotomus* (in the Old World) and *Lutzomyia* (in the New World). In Iran and other Old World regions, ZCL and ACL are predominantly caused by *Leishmania major* and *Leishmania tropica*, respectively (Golpayegani et al., 2018; Henry et al., 2021; Yaghoobi-Ershadi et al., 1996). The presence of the principal vectors, *Phlebotomus papatasi* for ZCL and *Phlebotomus sergenti* for ACL, has been well-documented throughout Iran, including Fars Province (Alipour et al., 2021; Azizi et al., 2016; Azizi et al., 2012a, Azizi et al., 2012b, Azizi et al., 2012c). Furthermore, gerbil rodents (Muridae: Gerbillinae), which are widely distributed in endemic areas, serve as the most important reservoir hosts for ZCL in Iran (Azizi et al., 2017; Azizi et al., 2012a, Azizi et al., 2012b, Azizi et al., 2012c; Motazedian et al., 2010).

Cutaneous leishmaniasis (CL) remains a significant public health concern in Iran and other endemic countries. In Fars Province, southern Iran, the incidence of CL has increased steadily over the past decade. Shiraz, a major tourist destination and population center, represents one of the principal foci of the disease. Continuous movement of immigrants and tourists into Shiraz further intensifies the risk of transmission and complicates control efforts. (Azizi et al., 2017).

This study provides an integrated entomological, molecular, and epidemiological assessment of *L. major* in Shiraz, Iran. By combining ITS2 genotyping, infection rate estimation, and ecological risk factor analysis, we aimed to characterize the zoonotic and environmental drivers influencing cutaneous leishmaniasis transmission in this endemic urban setting. Additionally, we analyzed the phylogeny and genetic structure of L. *major* using PCR targeting the kDNA region and sequencing of the ITS2 gene.

Although previous studies in Iran have employed kDNA and ITS2 markers for molecular characterization of *L. major*, limited studies have integrated entomological infection data, environmental context, and haplotype network analysis within a single emerging urban focus. The present study provides a combined epidemiological–molecular framework in Shiraz, contributing to a more comprehensive understanding of local transmission dynamics

## 2 Material and methods

### 2.1 Area of study

**Fig. 1:**
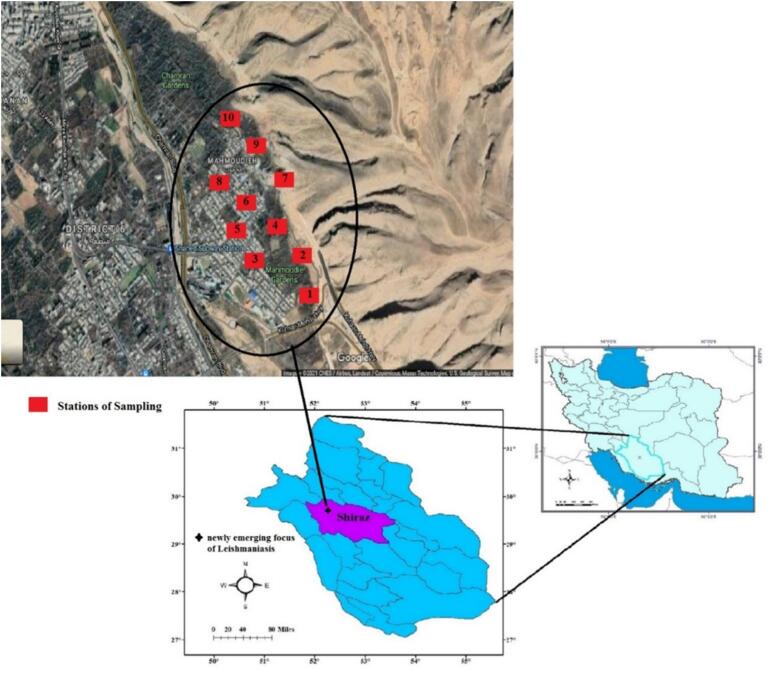
Map of Fars Province of Iran shows the sampling localities (stations 1 to 10) in the newly emerging focus of leishmaniasis in Shiraz, the fifth-most-populous city of Iran.

Fars Province, one of the largest in Iran, is situated in the southern part of the country (Fig. 1). Climatically, it is divided into four distinct regions: southern, western, central, and northern (Hatami and Khoushhal, 2010). The province encompasses the southernmost extent of the Zagros Mountain range, a region of significant zoogeographical importance that supports substantial animal diversity. Shiraz, the provincial capital (Fig. 1), is a major tourist destination and a key historical, cultural, social, industrial, and commercial center in southern Iran. It ranks as the country's third-largest city and fifth-most-populous urban area (Sarvestani et al., 2011). The city is located in a northwest-southeast oriented valley within the Zagros Mountains, at an average elevation of 1500 m above sea level. The northern skyline is dominated by the Baba Koohi and Ahmadi Heights, with the Sabz Pooshan Heights to the south and the Derak Heights to the west (Sarvestani et al., 2011). Shiraz experiences a moderate climate with four distinct seasons, where daily temperatures range from 40 °C in summer to 10 °C in winter. The city is renowned for its extensive gardens (Henry et al., 2021), and the surrounding mountainous and plain areas provide diverse habitats for a variety of animal and plant species.

### 2.2 Sand-fly sampling

Sand flies were collected indoors (e.g., bedrooms, bathrooms, and storage rooms) and outdoors within the city of Shiraz (Fig. 1) from August to October using sticky paper traps. Outdoor sampling sites were selected near potential breeding grounds, including running water, garbage dumps, wall crevices, and chicken coops in mountainous areas, as well as gardens adjacent to patients' residences. During each of the 20 sampling rounds, 50 sticky traps (25 indoors and 25 outdoors) were deployed after sunset. The traps were retrieved each morning, and the captured sand flies were carefully detached using entomological needles. The specimens were then washed with acetone and preserved in 70% ethanol for subsequent mounting and identification. Species identification was performed using established taxonomic keys based on external and internal morphological characteristics, including features of the head and posterior abdomen, male genitalia, and female spermathecae. After morphological identification, female sand flies were pooled into groups of six specimens per pool and stored in 70% ethanol for molecular analysis.

### 2.3 Patient sample collection and microscopic examination

Clinical samples were obtained from patients with cutaneous lesions, either during their visits to local health centers or through active field visits to their residences (Ethics Code: IR.SUMS.REC.1398.194). All samples were anonymized to protect patient confidentiality. Smears were prepared from the margins of the lesions, stained with Giemsa (Merck, Darmstadt, Germany), and examined microscopically for parasite detection. Subsequently, a random selection of 30 lesions from confirmed CL cases was analyzed by PCR to identify the causative *Leishmania* species.

### 2.4 DNA extraction

Pools of female sand flies were mechanically homogenized in 1.5 ml tubes using disposable pestles. Genomic DNA was then extracted from the homogenized pools and from patient lesion smears using the FavorPrep™ Tissue Genomic DNA Extraction Kit (Favorgen Biotech Corp; Cat. No. FABGK001) according to the manufacturer's protocol. The quantity and quality of the extracted DNA were assessed using a NanoDrop® 2000 spectrophotometer (Thermo Fisher Scientific, USA). Finally, the DNA was eluted in 50 μL of elution buffer and stored at −20 °C until further use.

### 2.5 Polymerase chain reaction (PCR)

For the kDNA gene, *Leishmania*-specific DNA was amplified using primers LIN4R (F: 5′- GGG GTT GGT GTA AAA TAG GG −3′) and LIN17 (R: 5′- TTT GAA CGG GAT TTC TG −3′). These primers generate fragments of approximately 650 bp for *L. major* and 760 bp for *L. tropica*. The PCR protocol consisted of an initial denaturation at 95 °C for 5 min; 35 cycles of denaturation at 94 °C for 30 s, annealing at 52 °C for 30 s, and extension at 72 °C for 45 s; followed by a final extension at 72 °C for 8 min. Amplifications were performed in an Eppendorf thermal cycler (Hamburg, Germany).

The ITS2 gene was amplified using the primers 5′-AAACTCCTCTCTGGTGCTTGC-3′ (forward) and 5′-AAACAAAGGTTGTCGGGGG-3′ (reverse). Each 25 μL reaction contained 1 μL of extracted DNA (100 ng/μL), 0.6 μL of each primer (10 pmol/μL), 12.5 μL of 1× Taq DNA Polymerase Master Mix RED, and 10.3 μL of distilled water. The thermal cycling conditions were: initial denaturation at 94.5 °C for 5 min; 35 cycles of 94 °C for 30 s, 55 °C for 30 s, and 72 °C for 30 s; with a final extension at 72 °C for 8 min.

PCR products (3.5 μL) were separated alongside a 100-bp DNA ladder (SMOBIO, Hsinchu, Taiwan) on a 2% agarose gel (Sigma Aldrich, USA). The gel was stained with SYBR® Safe DNA Gel Stain (Thermo Fisher Scientific, Carlsbad, CA, USA), electrophoresed at 80 V for 45 min, and visualized under UV trans-illumination (Uvitec, Cambridge, UK). Reference strains of *L. major* (MHOM/IR/54/LV39) and *L. tropica* (MHOM/IR/89/ARD-L2) served as positive controls, while distilled water (DW) was used as a negative control.

### 2.6 Phylogenetic analyses

The raw nucleotide sequences from both forward and reverse directions were inspected and analyzed using the Chromas program within BioEdit version 7.2.5 (Hall, 1999). Consensus sequences were generated and aligned using the Clustal W algorithm implemented in the software. The resulting twelve sequences were deposited in the GenBank database under accession numbers ON398772–ON398780 and ON398788–ON398791. The final alignment, comprising 482 base-pair positions, was converted into FASTA format for subsequent analysis and also exported in NEXUS format using MEGA X software (Kumar et al., 2018). These consensus sequences were compared to homologous sequences in the GenBank database via the BLAST algorithm (Camacho et al., 2009). For the phylogenetic and genetic structure analyses, we included the 12 partial ITS2 sequences generated in this study, 46 published sequences from our previous work (Shahabi et al., 2024), and additional sequences retrieved from GenBank (Fig. 4) originating from Western Asia (Iran, Iraq, Turkmenistan), Western Europe (Spain, Portugal), and Africa (Kenya, Sudan, Mali).

Phylogenetic relationships between *Leishmania* species were reconstructed using Bayesian inference (BI) in BEAST v2.6.7. The Markov chain Monte Carlo (MCMC) analysis was run for 10 million generations, with trees sampled every 1000 generations. Node support was assessed using Bayesian posterior probabilities. Additionally, a Neighbor-Joining (NJ) tree was constructed in MEGA X using the Kimura 2-parameter (K2P) model, with branch support evaluated via 10,000 bootstrap replicates. Support values from both analyses (posterior probabilities and bootstrap percentages) are shown on the respective tree nodes.

A haplotype network based on the ITS2 gene sequences was constructed using the TCS algorithm in PopART (Population Analysis with Reticulate Trees) (Leigh and Bryant, 2015). We assessed the molecular diversity of 58 *L. major* ITS2 sequences from this study and GenBank using DnaSP version 6.0 (Librado and Rozas, 2009). The summary statistics calculated included the total number of sites, number of haplotypes, number of polymorphic sites, average number of nucleotide differences, nucleotide diversity, haplotype diversity, and the number of parsimony informative sites.

### 2.7 Statistical analysis

Female sand flies were pooled into groups of six individuals, resulting in 20 pools for *Ph. papatasi* (*n* = 120) and 20 pools for *Ph. sergenti* (n = 120). A pool was defined as positive if at least one sand fly contained *Leishmania* DNA. Consequently, infection prevalence is reported as the Minimum Infection Rate (MIR), a conservative estimate that assumes a single infected sand fly per positive pool. The MIR per 1000 sand flies was calculated as (number of positive pools / total number of individual sand flies tested) × 1000. Differences in infection rates between the two *Phlebotomus* species were assessed using Fisher's exact test, chosen due to the limited number of pools. A *p*-value of less than 0.05 was considered statistically significant. All analyses were performed using SPSS software (Version 26).

## 3 Results

### 3.1 Environmental observation

**Fig. 2:**
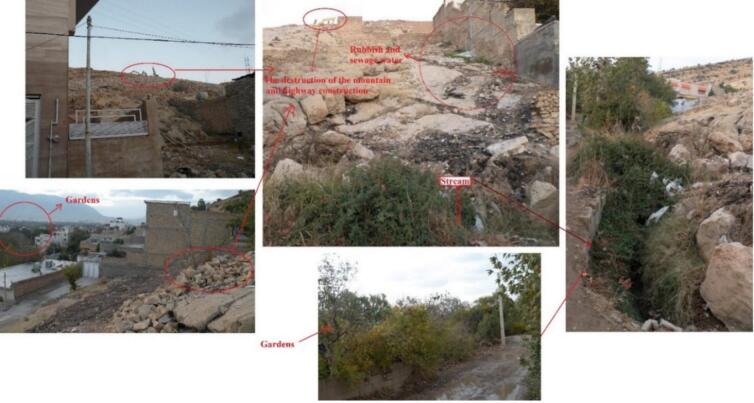
Sampling area showing the environmental factors including stream, sewage water, rubbish, garden, and mountainous habitats, destruction of Mountain and highway construction in the city Shiraz. These factors are associated with conditions that may support the presence, reproduction, and distribution of sand flies.providing suitable habitat for reservoirs and vectors of *Leishmania* causing cutaneous leishmaniasis in the city.

The study area (Fig. 1, Fig. 2) encompasses foothills, orchards—primarily of walnut and pomegranate trees—and residential zones. Environmental observations revealed ongoing mountain excavation for road construction, the dumping of domestic refuse adjacent to buildings, and the discharge of household sewage into open water channels. The presence of domestic animals, such as chickens and dogs, was also noted within the residential areas.

### 3.2 Clinical patient analysis

**Fig. 3:**
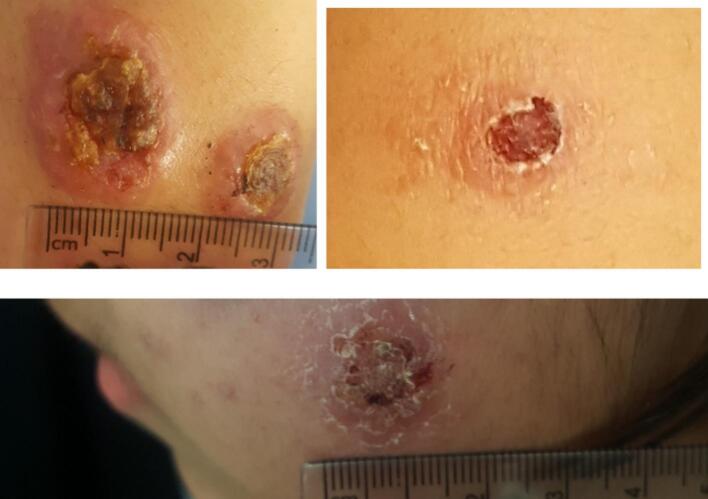
Lesions caused by *Leishmania* species (*L. tropica* and *L. major*) confirmed by PCR of kDNA and ITS2 Gene.

**Fig. 4:**
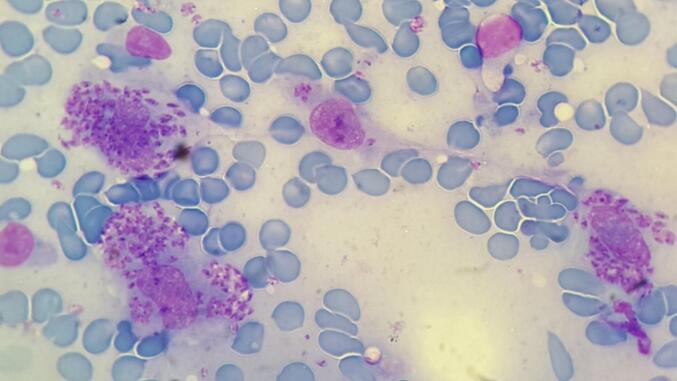
*Leishmania* amastigotes within the disintegrated macrophage (Giemsa staining, 1000 × magnification) of a Leishmaniasis patient.

**Fig. 5:**
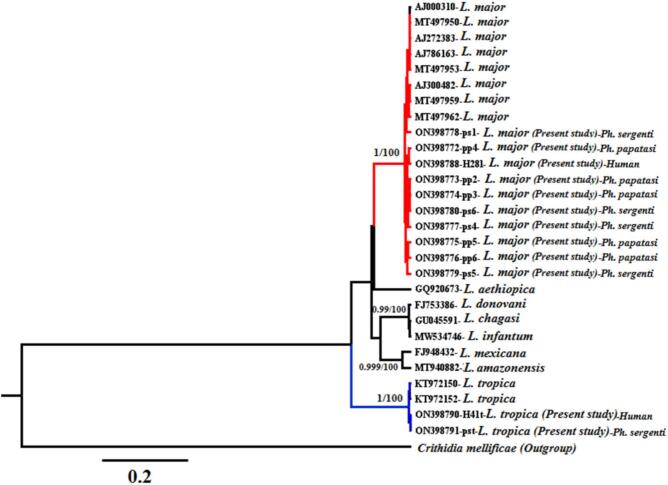
The evolutionary relationships of *Leishmania* species infecting human lesions and sand flies sampled in the present study based on the DNA sequences of ITS2 gene inferring based on the Bayesian method. The posterior probability value for the Basian tree and the percentage value of bootstrap tests (10,000 replicates) for the Neighbor-Joining tree are shown at the base of the clades before and after the slash symbol, respectively.

All 30 randomly selected patients were confirmed to have CL through both microscopic examination and PCR analysis of the kDNA gene (Fig. 3, Fig. 4). More than half of the patients living in the foothills and garden areas were found to be infected with *L. major*. In contrast, those living in areas distal to the mountains, near the city's main Chamran Boulevard, were infected with *L. tropica*. The species identity in two representative lesion samples from ZCL and ACL patients was further confirmed by genetic sequencing and phylogenetic analysis of the ITS2 region (Fig. 4, Fig. 5).

### 3.3 Sand fly fauna and positive samples for *Leishmania*

A total of 1029 sand flies were collected using sticky paper traps. Of these, 763 (74.15%) were male and 266 (25.85%) were female. Specimens were collected from both indoor (542, 52.7%) and outdoor (487, 47.3%) locations. Morphological identification classified all specimens into two genera: *Phlebotomus* and *Sergentomyia*.

Among the 266 female sand flies identified, *Ph. papatasi* was the most prevalent species (143; 53.75%), followed by *Phlebotomus sergenti* (103; 38.75%). Less abundant species included *Sergentomyia dentata* (6; 2.25%), *Ph. bergeroti* (4, 1.5%), and *Sergentomyia* sp. (10, 3.75%).

The collection data revealed a strong indoor preference for the primary species: 86% of female *Ph. papatasi* (*n* = 151) and 56% of female *Ph. sergenti* (*n* = 58) were captured indoors. Consequently, *Ph. papatasi* was designated the dominant species in the study area, with *Ph. sergenti* as the second most dominant, and *S. dentata* as the third.

Of the 40 pools tested, *Leishmania* DNA was detected in 16 (80%) of the *Ph. papatasi* pools and 13 (65%) of the *Ph. sergenti* pools, resulting in an overall infection rate of 72.5%. The Minimum Infection Rate (MIR) was 112/1000 for *Ph. papatasi*, 126/1000 for *Ph. sergenti*, and 118/1000 overall. Fisher's exact test revealed no significant difference in infection rates between the two species (odds ratio = 2.15, *p* = 0.48). Although a majority of positive pools were collected indoors, the absence of precise stratification data precluded a statistical comparison of infection prevalence by collection site.

All pools of *Ph. papatasi* and nine pools of *Ph. sergenti* tested positive for *L. major* DNA. In contrast, four other pools of *Ph. sergenti* were positive for *L. tropica* DNA. No other sand fly species were infected. The majority of positive sand flies were collected indoors—including bedrooms, kitchens (near rodent burrows), and storage rooms located directly above bathrooms—as well as from chicken coops.

### 3.4 Phylogenetic analysis

Phylogenetic analysis of the ITS2 sequences corroborated the kDNA PCR results, confirming the detection of both *Leishmania* species—*L. major* and *L. tropica*—in both patient lesions and *Phlebotomus* sand flies. Bayesian and Neighbor-Joining analyses showed that *Leishmania* parasites from patients and sand flies clustered into two main monophyletic clades (Fig. 5). These clades received strong statistical support, with 100% bootstrap values and posterior probabilities, as indicated at the base of each clade. The mean nucleotide diversity within the *L. major* clade was 0.01.

### 3.5 Genetic structure and haplotype network

Analysis of a 482-bp fragment of the ITS2 gene across all 58 sequences identified 28 polymorphic sites, including nine singleton variable sites and 19 parsimony-informative sites, which collectively defined 22 unique haplotypes (Fig. 4). Overall, the population exhibited a nucleotide diversity (π) of 0.01 and a high haplotype diversity (Hd) of 0.74.

**Fig. 6:**
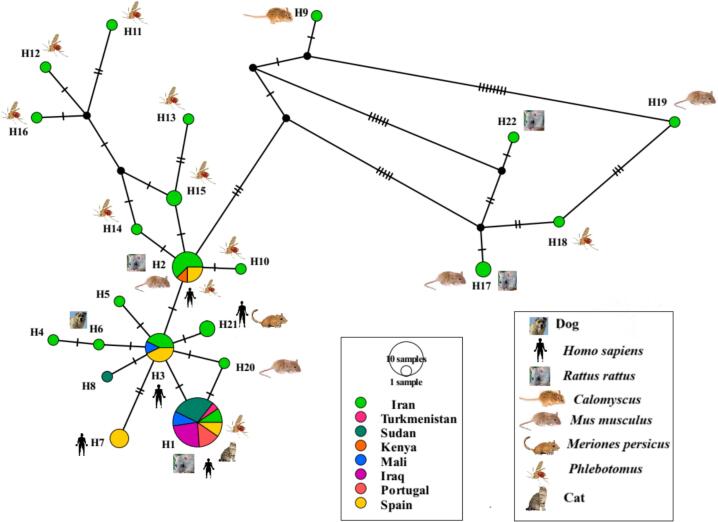
Haplotype network obtained for 482 bp of 58 ITS2 sequences of *L. major* from the present study and Gene Bank (NCBI) circulating in human and various animals. Circle size is relative to haplotype frequency. Black circles represent extinct or unsampled haplotypes. Hatch marks on the line represent mutational steps between haplotypes. Haplotype colors represent geographic locations of haplotypes indicated on the right side of the figure.

**Fig. 7:**
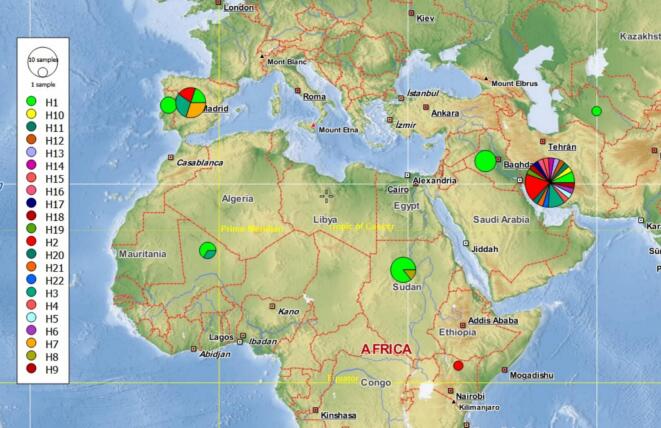
A geographical distribution of *L. major* haplotypes present in Western Asia, Africa and Western Europe. Each color represents a haplotype. Circle size is relative to haplotype frequency. Position of circles within a country does not indicate a certain locality.

Of the 22 identified haplotypes, 20 were found in Iran (Fig. 6; Table 1). Only three of these were shared between Iran and other countries in Asia, Africa, and Europe (Fig. 7). Among the Iranian ITS2 haplotypes identified in this study, 12 corresponded to previously reported haplotypes documented in GenBank, while 8 haplotypes are reported here for the first time. Haplotype 1 was the most frequent (*n* = 21) and ancestrally dominant, being shared across all sampled countries in Asia, Africa, and Europe with the exception of Kenya (Fig. 6, Fig. 7; Table 1). Iran harbored 17 distinct haplotypes, which were newly identified in this study. These were distributed across different hosts: eight were found in sand flies, five in rodents, and four in humans and other animals. The highest values of both haplotype and nucleotide diversity were observed in the sequences from Iran (Table 1).

**Table 1:** Summary statistics of molecular diversity within ITS2 sequences of *L. major*; Total number of sites (excluding sites with gaps / missing data) were 482. n – number of individuals, h – number of haplotypes, S- number of polymorphic (segregating) sites, K- average number of nucleotide differences, Pi – nucleotide diversity, Hd – haplotype diversity, Hap-Haplotype.

	Western Asia			Africa			Western Europe		Total
	Iran	Turkmenistan	Iraq	Sudan	Kenya	Mali	Portugal	Spain	
n	30	1	5	7	1	2	3	10	59
h	20	1	1	2	1	1	1	4	22
S	20	–	–	1	–	–	–	4	46
K	5.88	–	–	0.286	–	–	–	1.29	3.75
Pi	0.016	–	–	0.0008	–	–	–	0.0035	0.01
Hd	0.924	–	–	0.286	–	–	–	0.69	0.74
Hap1	2	1	5	6	0	2	3	2	21
Hap2	5	0	0	0	1	0	0	2	8
Hap3	3	0	0	0	0	0	0	3	6
Hap4	1	0	0	0	0	0	0	0	1
Hap5	1	0	0	0	0	0	0	0	1
Hap6	1	0	0	0	0	0	0	0	1
Hap7	0	0	0	0	0	0	0	3	3
Hap8	0	0	0	1	0	0	0	0	1
Hap9	1	0	0	0	0	0	0	0	1
Hap10	1	0	0	0	0	0	0	0	1
Hap11	1	0	0	0	0	0	0	0	1
Hap12	1	0	0	0	0	0	0	0	1
Hap13	1	0	0	0	0	0	0	0	1
Hap14	1	0	0	0	0	0	0	0	1
Hap15	2	0	0	0	0	0	0	0	2
Hap16	1	0	0	0	0	0	0	0	1
Hap17	2	0	0	0	0	0	0	0	2
Hap18	1	0	0	0	0	0	0	0	1
Hap19	1	0	0	0	0	0	0	0	1
Hap20	1	0	0	0	0	0	0	0	1
Hap21	2	0	0	0	0	0	0	0	2
Hap22	1	0	0	0	0	0	0	0	1

## 4 Discussion

This study documents ongoing ZCL and ACL transmission in Shiraz, which appears to be associated with peridomestic sand flies (*Ph. papatasi* and *Ph. sergenti*) and local environmental conditions. Environmental mismanagement—such as garbage accumulation near homes, open sewage, and proximity to animals—was associated with higher sand fly presence.The spread and emergence of the disease are influenced by a combination of zoonotic and environmental drivers (Charrahy et al., 2022; Firooz et al., 2021; Sabzevari et al., 2021; Shahabi et al., 2024), with vector abundance being a key contributor. The high *Leishmania* infection rate in sand flies (72.5%) further indicates intense zoonotic circulation in the region.

The high proportion of *Ph. papatasi* collected indoors (86%) underscores substantial human–sand fly contact within households, reinforcing its role as the principal vector of *L. major* in Iran and other regions of Southwest Asia and North Africa (Elaagip et al., 2020). Environmental mismanagement, such as the disposal of garbage near residences, appears to be associated with increased vector presence.

The clustering of ZCL cases near foothills and gardens is consistent with the known habitats of rodent reservoirs (Shahabi et al., 2023, Shahabi et al., 2024), while the occurrence of ACL in urban areas suggests a primarily anthroponotic transmission cycle.

Although Fisher's exact test revealed no significant difference in infection rates between *Ph. papatasi* and *Ph. sergenti*, the statistical power of this finding may be limited by the relatively small sample size. Nonetheless, the detection of *L. major* and *L. tropica* in these species confirms that both ZCL and ACL are circulating in the region. This underscores the critical role of both *Ph. papatasi* and *Ph. sergenti* as primary vectors in the local transmission cycles of cutaneous leishmaniasis.

*L. tropica* was more frequently detected in *Ph. sergenti* in Iran (Azizi et al., 2012a, Azizi et al., 2012b, Azizi et al., 2012c; Azizi et al., 2016; Yaghoobi-Ershadi, 2012). In the present study, *L. major* DNA was also identified in a majority of blood-engorged *Ph. sergenti* pools (9 out of 20). This is likely due to the species feeding on ZCL patients, as the PCR detection of parasite DNA indicates exposure but does not confirm vector competence. Detection of parasite DNA in *this species* does not establish its role as a competent vector; experimental transmission studies would be required to confirm vector competence.

No *Leishmania* infection was observed in *Ph. bergeroti* in our study. However, the involvement of *P. bergeroti* in the transmission of *L. major* has been reported in Iran (Azizi et al., 2016; Parvizi et al., 2013) and other regions, including Ethiopia (Seccombe, 1993), Kenya (Balkew et al., 2002), and the Sahara (Beach et al., 1984). Therefore, future studies should investigate the role of such secondary vectors in the region. However, sampling period (August–October) may not fully represent seasonal variation and could bias infection rate estimates.

The ITS2 haplotype network revealed that Iran harbors the highest global diversity of *L. major* haplotypes, with 20 of the 22 identified haplotypes found there, 17 of which were unique to the country. The substantial genetic diversity observed among Iranian *L. major* isolates (Hd = 0.924) is consistent with previous findings, which have reported similar heterogeneity using both the ITS2 marker (Nikookar et al., 2024; Shahabi et al., 2024) and alternative genetic markers (Kalantari et al., 2025; Mahmoudzadeh-Niknam et al., 2012; Mahnaz et al., 2011; Nemati et al., 2024; Sarkari et al., 2016). This exceptional genetic diversity indicates that the region is a long-standing evolutionary hotspot for *L. major*. This is likely driven by persistently favourable ecological conditions that maintain diverse vector (e.g., *Ph. papatasi*, *Ph. sergenti*) and reservoir (e.g., gerbil) populations (Kalantari et al., 2025; Shahabi et al., 2024). Such high genetic variability likely reflects long-term circulation of diverse strains in the region. However, ITS2 variation alone does not indicate differences in virulence, pathogenicity, or drug resistance. The dispersal of infected sand flies and reservoir hosts from this region poses a substantial risk for the global spread of *Leishmania*, as demonstrated by the ancestral Hap1 shared across Asia, Africa, and Europe. Consequently, Iran constitutes a key hotspot for the emergence and dissemination of this parasite. It should be noted, however, that the observed ITS2 diversity could be influenced by intra-genomic variation among multiple gene copies, meaning it cannot reliably predict speciation, virulence, or drug resistance. Furthermore, the ITS2 marker provides limited resolution for deep phylogenetic relationships. Therefore, employing multilocus approaches—such as microsatellites, multilocus sequence typing (MLST), or kDNA analysis—would provide valuable complementary data.

Iranian *Leishmania* isolates demonstrated considerable diversity within the global ITS2 dataset, contributing substantially to overall haplotype variation. This observation highlights Iran as an important region for genetic diversity of *L. major*. However, although ITS2 is a useful marker for population- and species-level differentiation, it has limited resolution for deep phylogenetic inference. Therefore, conclusions regarding evolutionary relationships should be interpreted cautiously.

Another factor associated with the emergence of ACL and ZCL is habitat destruction caused by highway construction. This environmental change may influence rodent movement and contribute to the spread of ZCL toward urban areas. The resultant disturbed environment, particularly unattended water sources, attracts stray dogs and other mammals. Furthermore, the peridomestic presence of animals such as domestic dogs and chickens in households and gardens provides abundant blood meal sources. Female sand flies, which require blood for egg development, feed on a variety of hosts including rodents, humans, livestock, dogs, and chickens (Ramalho-Ortigão et al., 2007; Sant'Anna et al., 2008). The availability of these hosts supports the proliferation and survival of the primary vectors, *Ph. papatasi* and *Ph. sergenti*, enabling them to acquire and transmit *Leishmania* species between humans and other animals.

Compounding these issues, the improper discharge of domestic sewage into surface waters used to irrigate nearby orchards may create additional sites suitable for sand fly breeding and feeding. Along with garbage accumulation, wastewater mismanagement, abundant orchards, rodent presence, construction debris, and high population density (Fig. 2), these environmental conditions appear to support the presence, reproduction, and distribution of sand fly vector species. Therefore, integrated measures—including rodent control, restricting the proximity of domestic animals to human dwellings, and public education on personal protection against sand fly bites—are strongly recommended. (Desjeux, 2001; Oryan and Akbari, 2016; Ximenes, 2009).

## 5 Conclusion

Shiraz remains a high-risk focus for cutaneous leishmaniasis due to a combination of ecological factors (vector and reservoir abundance), environmental conditions (poor sanitation), and genetic factors (high *L. major* diversity). Implementing integrated control measures—including vector reduction, improved waste management, and community education—is crucial. Genetic monitoring can help track emerging strains and their zoonotic potential, and investigating haplotype-specific differences in pathogenicity is recommended. These findings underscore the importance of continued surveillance and integrated vector management strategies in urban and peri-urban settings.

## CRediT authorship contribution statement

**Kourosh Azizi:** Writing – review & editing, Methodology, Investigation, Conceptualization. **Saeed Shahabi:** Writing – review & editing, Writing – original draft, Project administration, Investigation, Conceptualization. **Bahador Sarkari:** Writing – review & editing, Validation, Formal analysis. **Qasem Asgari:** Writing – review & editing, Validation, Methodology. **Aboozar Soltani:** Validation, Software, Methodology. **Mohsen Kalantari:** Writing – review & editing, Methodology, Investigation. **Azim Paksa:** Writing – review & editing, Software, Investigation. **Sorna Dabaghmanesh:** Writing – review & editing, Formal analysis, Data curation.

## Consent for publication

All authors have read and approved the final manuscript and provide their consent for its publication in *Parasites Epidemiology and Control*. We confirm that this work is original and is not under consideration for publication elsewhere.

## Ethics approval and consent to participate

This study was approved by the Ethics Committee of Shiraz University of Medical Sciences (number: IR.SUMS.REC.1398.194). The authors have no competing interests to declare.

## Funding

This work was supported by the 10.13039/501100003968Iran National Science Foundation (INSF) [Postdoctoral Project No. 99002571] and the Vice-Chancellorship for Research and Technology of Shiraz University of Medical Sciences (SUMS) [Grant No. 97-01-106-18847].

## Declaration of competing interest

The authors of the present study declare no conflict of interests.

## Data Availability

The ITS2 gene sequences generated in this study are available in the GenBank database under accession numbers ON398772–ON398780 and ON398788–ON398791.
